# Development and validation of a dynamic nomogram based on conventional ultrasound and contrast-enhanced ultrasound for stratifying the risk of central lymph node metastasis in papillary thyroid carcinoma preoperatively

**DOI:** 10.3389/fendo.2023.1186381

**Published:** 2023-06-20

**Authors:** Qiyang Chen, Yujiang Liu, Jinping Liu, Yuan Su, Linxue Qian, Xiangdong Hu

**Affiliations:** Department of Ultrasound, Beijing Friendship Hospital, Capital Medical University, Beijing, China

**Keywords:** contrast-enhanced ultrasound (CEUS), papillary thyroid carcinoma, central lymph node metastasis, risk assessment, dynamic nomogram

## Abstract

**Purpose:**

The aim of this study was to develop and validate a dynamic nomogram by combining conventional ultrasound (US) and contrast-enhanced US (CEUS) to preoperatively evaluate the probability of central lymph node metastases (CLNMs) for patients with papillary thyroid carcinoma (PTC).

**Methods:**

A total of 216 patients with PTC confirmed pathologically were included in this retrospective and prospective study, and they were divided into the training and validation cohorts, respectively. Each cohort was divided into the CLNM (+) and CLNM (−) groups. The least absolute shrinkage and selection operator (LASSO) regression method was applied to select the most useful predictive features for CLNM in the training cohort, and these features were incorporated into a multivariate logistic regression analysis to develop the nomogram. The nomogram’s discrimination, calibration, and clinical usefulness were assessed in the training and validation cohorts.

**Results:**

In the training and validation cohorts, the dynamic nomogram (https://clnmpredictionmodel.shinyapps.io/PTCCLNM/) had an area under the receiver operator characteristic curve (AUC) of 0.844 (95% CI, 0.755–0.905) and 0.827 (95% CI, 0.747–0.906), respectively. The Hosmer–Lemeshow test and calibration curve showed that the nomogram had good calibration (*p* = 0.385, *p* = 0.285). Decision curve analysis (DCA) showed that the nomogram has more predictive value of CLNM than US or CEUS features alone in a wide range of high-risk threshold. A Nomo-score of 0.428 as the cutoff value had a good performance to stratify high-risk and low-risk groups.

**Conclusion:**

A dynamic nomogram combining US and CEUS features can be applied to risk stratification of CLNM in patients with PTC in clinical practice.

## 1 Introduction

Papillary thyroid carcinoma (PTC) is the most common type of thyroid cancer, accounting for 80%–90% of all thyroid carcinomas (1, 2). PTC is a lymphotropic tumor, and 20%–90% of patients with PTC develop cervical lymph node metastasis (LNM), and approximately 70% of these cases involve central lymph node metastasis (CLNM) (3, 4). For patients with PTC, CLNM is an important risk factor for distant metastasis or tumor recurrence and an indicator for surgical strategy (5–8); in these cases, central compartment lymph node dissection (CLND) is recommended (9). However, preoperative identification of CLNM has been a challenge. Approximately 45% of PTC patients with clinically negative central ventricular LNs (cN0) were reported to have CLNM pathologically confirmed after surgery (10). Whether prophylactic CLND should be performed in cN0 PTC patients is still under debate, possibly raising risks of nerve injury and hypoparathyroidism (11). Therefore, accurate and noninvasive preoperative prediction of CLNM has been of increasing importance in clinical practice to optimize treatment decisions.

Conventional ultrasound (US) is the first-line modality for evaluating thyroid nodules and cervical lymph nodes (9). However, US is limited in detecting CLNM because of interference of the thyroid gland and adjacent organs. As reported, just 30.0%–53.2% of cases with CLNM could be detected by US (2, 12, 13). In recent years, some US-based imaging modalities were proposed to enhance the capability of identifying CLNM. A radiomics nomogram based on the shear wave elastography (SWE) was established by Jiang et al. (7). However, SWE has not been widely used in clinical practice.

Contrast-enhanced US (CEUS) is an imaging modality that reveals tumor microvascular perfusion through the accumulation of contrast agent microbubbles in blood vessels (14). CEUS has been widely applied to distinguish benign and malignant thyroid nodules (15, 16). Several studies reported that CEUS may be a potential tool to predict CLNM in patients with PTC (14, 17). However, most studies focused on CEUS features alone and failed to provide a feasible and generalizable prediction model.

In this study, we aimed to develop and validate a nomogram by combining US and CEUS features to facilitate preoperative risk stratification for individualizing treatment decision.

## 2 Materials and methods

### 2.1 Patients

This two-way cohort study was approved by the Ethics Committee of our hospital. The requirement for individual consent for retrospective data was waived. All patients with prospective data signed informed consent.

Patients who underwent total or partial (lobectomy or near-total thyroidectomy) thyroidectomy for PTC between January 2017 and December 2019 were retrospectively evaluated from the institutional database. From February 2021 to October 2021, we prospectively recruited PTC patients who were diagnosed by ultrasound-guided fine needle aspiration and prepared to receive surgery in our institution. Patients were enrolled according to the following inclusion criteria (1): solitary PTC was determined pathologically; (2) CLND was performed; and (3) US and CEUS were performed preoperatively. The exclusion criteria were as follows: (1) maximum tumor diameter was <5 mm (CEUS is limited by respiratory motion and volume effect); (2) US or CEUS image was incomplete or unclear; (3) skip metastases were found; and (4) history of secondary malignancy.

**Figure 1:**
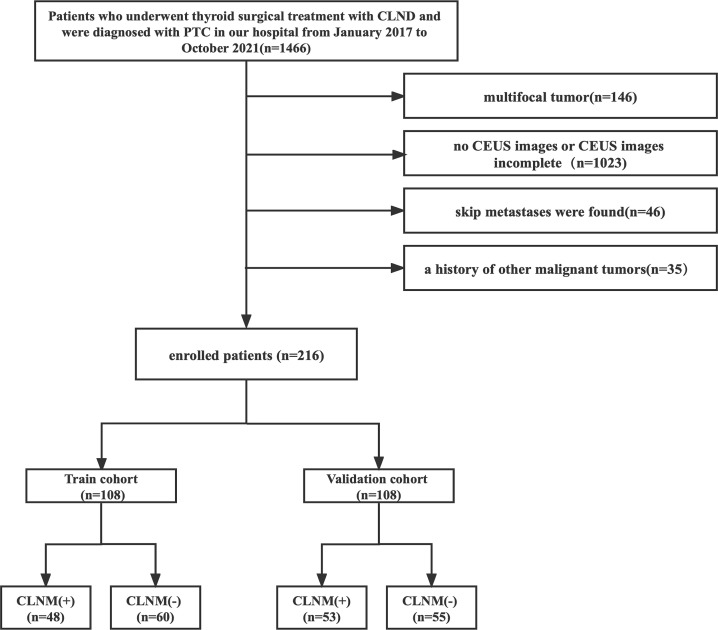
Flowchart of patient enrollment in the study.

A total of 216 patients with pathologically confirmed PTC were included. The patients were divided into the training and validation cohorts, with a patient ratio in the training to validation cohorts of 1:1. The training cohort consisted of 108 patients (33 male and 75 female patients; mean age, 43.72 ± 12.44 years), and the validation cohort enrolled 108 patients (20 male and 88 female patients; mean age, 44.4 ± 11.48 years) who were randomly selected from the prospective data. Each cohort was divided into CLNM (+) and CLNM (−) groups according to the pathology results. **Figure 1** shows the patient selection.

### 2.2 US and CEUS image acquisition

Training cohort images were acquired with an EPIQ5 (Philips Ultrasound, Inc., Bothell, Washington, USA) system equipped with a 5- to 12-MHz linear probe, and validation cohort images were acquired with a Resona7 (Shenzhen Mindray Bio-Medical Electronics Co., Ltd., Shenzhen, China) system equipped with a 5- to 12-MHz linear probe. US and CEUS images for individuals were obtained from the same instruments. SonoVue (Bracco, SpA, Milan, Italy) was used for all patients as a contrast agent with a mechanical index of 0.08 and a volume of 1.2–2.4 ml (2.4 ml in the validation cohort) when CEUS was performed. The US features of the lesion were carefully evaluated, including size, location, margin, echogenicity, aspect ratio, calcification, and contact with the capsule. The longitudinal plane with the clear lesion was selected for CEUS. The contrast agent was injected intravenously as a bolus, followed by a 5-ml saline flush (0.9% sodium chloride). All CEUS images were observed for 3 min and stored on the hard disk for further analysis.

The US and CEUS features were independently analyzed by two experienced radiologists (with more than 10 years of experience in thyroid imaging) blinded to pathological outcomes. If the radiologists disagreed, a consensus was obtained by discussion.

### 2.3 Conventional US image analysis

US features were characterized as follows. Size refers to the maximum tumor diameter. The location was classified into the left lobe, right lobe, and isthmus. The margin was divided into regular or irregular. Echogenicity was classified into hypoechoic, isoechoic, or hyperechoic relative to surrounding thyroid parenchyma. The aspect ratio was classified as ≤1 or >1. Contact with the capsule was described as yes or no according to whether more than 20% of the tumor was touching the thyroid capsule or an absence of echogenicity of the thyroid capsule on US.

### 2.4 CEUS image analysis

The CEUS qualitative parameters were defined as follows: (1) enhancement patterns (centripetal or hybrid enhancement), (2) homogeneity of enhancement (homogeneous or heterogeneous), (3) enhancement intensity (hypo-enhancement, iso-, or hyperenhancement compared with surrounding thyroid parenchyma), (4) time of wash-in (earlier or concurrent and later), (5) time of wash-out time (earlier or concurrent and later), and (6) discontinuous capsular enhancement (anterior and/or posterior hyperechoic thyroid capsular was discontinued).

The CEUS quantitative parameters were obtained by QLAB or Resona7 system software. The region of interest (ROI) was first outlined along the outer margin of the tumor, defined as ROI1. ROI2 was copied from ROI1 and outlined in the surrounding thyroid parenchyma at the same tumor depth. Two time–intensity curves (TIC) were obtained. The analysis time was the first 60 s of the dynamic CEUS images. TIC parameters included the following: (1) wash in slope (WIS), (2) time to peak (TP), (3) peak intensity (PI), and (4) area under the curve (AUC). These values were measured three times for each tumor and averaged as P_ROI1_ and P_ROI2_. The ratio of P_ROI1_ to P_ROI2_ (P_ROI1_/_ROI2_) was used for comparison to reduce the potential effect of differences from the imaging system, image analysis software, and contrast agent doses.

### 2.5 Feature selection and model construction

The least absolute shrinkage and selection operator (LASSO) regression with penalty parameter tuning conducted by 10-fold cross-validation was applied in the training cohort to select useful predictive features for CLNM. Univariate and multivariate logistic analyses were performed to identify the risk factors of CLNM. The prediction model was established by combining LASSO and multivariate logistic regression analysis and presented in the form of a nomogram.

### 2.6 Evaluation and validation of the model

The prediction model’s performance was evaluated by receiver operating characteristic (ROC) curves in the training and validation cohorts. AUC was calculated to assess the discrimination performance of the prediction model in the training and validation cohorts. Calibration of the nomogram was evaluated using the calibration curve and Hosmer–Lemeshow test.

### 2.7 Clinical utility of the prediction model

To estimate the predictive value of the prediction model, decision curve analysis (DCA) was performed by quantifying the net benefits at different threshold probabilities in the validation cohort.

The Nomo-score, that is, the nomogram-predicted probability, was calculated in each patient. The cutoff value of the Nomo-score was obtained through the maximum Youden index, and patients were classified as high risk and low risk using this value.

### 2.8 Statistical analysis

Statistical analysis was conducted with SPSS Statistics version 26.0 (IBM Corp.), R software version 4.1.0 (The R Foundation for Statistical Computing), and GraphPad Prism 9.0. Quantitative data were presented as mean ± standard deviation, and Mann–Whitney *U* tests were used for comparison. Categorical data were presented as numbers and percentages, and the chi-square test was used for comparison. The Delong test was used in ROC. The two-sided *p* < 0.05 was considered as significant difference.

## 3 Results

### 3.1 Patient characteristics

Clinical characteristics, US, and CEUS features of all patients are summarized in **Table 1**. Except for margin, aspect ratio, and time of wash-in, there were no differences in characteristics between the two cohorts (*p* > 0.05). The rates of CLNM were 44.4% (48/108) and 49.1% (53/108) in the training and validation cohorts, respectively, with no significant difference found between the cohorts (*p* = 0.495).

**Table 1:** Characteristics of patients in the training and validation cohorts.

Characteristics	Training cohort (*N* = 108)	Validation cohort (*N* = 108)	*p*-value
CLNM			0.495
CLNM (+)	48 (44.4%)	53 (49.1%)	
CLNM (−)	60 (55.6%)	55 (50.9%)	
Sex			0.946
Male	22 (20.4%)	20 (18.5%)	
Female	86 (79.6%)	88 (81.5%)	
Age (years)	43.6 ± 12.3	44.4 ± 11.48	0.554
>42	51 (47.2%)	55 (50.9%)	0.586
≤42	57 (52.8%)	53 (49.0%)	
Size (cm)	1.09 ± 0.53	1.04 ± 0.54	0.558
≥0.95	61 (56.5%)	48 (44.4%)	0.077
<0.95	47 (43.5%)	60 (55.6%)	
Location			0.118
Left lobe	49 (45.3%)	61 (56.5%)	
Right lobe	57 (45.3%)	47 (43.5%)	
Isthmus	2 (45.3%)	0	
Margin			<0.001
Regular	40 (37.0%)	14 (13.0%)	
Irregular	68 (63.0%)	94 (87.0%)	
Aspect ratio			0.021
>1	63 (58.3%)	46 (42.6%)	
≤1	45 (41.7%)	62 (57.4%)	
Calcification			0.111
Yes	87 (80.6%)	77 (71.3%)	
No	21 (19.4%)	31 (28.7%)	
Echogenicity			0.358
Hypoechoic	99 (91.7%)	104 (96.3%)	
Isoechoic	7 (6.5%)	3 (2.8%)	
Hyperechoic	2 (1.9%)	1 (0.9%)	
Contact with the capsule			0.761
Yes	79 (73.1%)	77 (71.3%)	
No	29 (26.9%)	31 (28.7%)	
Enhancement intensity			1.000
Hypo-enhancement	61 (56.5%)	61 (56.5%)	
Iso- or hyperenhancement	47 (43.5%)	47 (43.5%)	
Enhancement patterns			0.575
Centripetal enhancement	65 (60.2%)	69 (63.9%)	
Hybrid enhancement	43 (39.8%)	39 (36.1%)	
Homogeneity of enhancement			0.390
Homogeneous	34 (31.5%)	40 (37.0%)	
Heterogeneous	74 (68.5%)	68 (63.0%)	
Discontinuous capsular enhancement			0.118
Yes	33 (30.6%)	44 (40.7%)	
No	75 (69.4%)	64 (59.3%)	
Time of wash-in			<0.001
Earlier	78 (72.2%)	13 (12.0%)	
Meantime and later	30 (27.8%)	95 (88.0%)	
Time of wash-out time			0.122
Earlier	35 (32.4%)	46 (42.6%)	
Meantime and later	73 (67.6%)	62 (57.4%)	
WIS	1.05 ± 0.97	0.87 ± 0.43	0.341
TP	1.10 ± 0.43	1.16 ± 1.16	0.698
PI	0.82 ± 0.29	0.77 ± 0.33	0.176
AUC	0.82 ± 0.33	0.75 ± 0.36	0.115

### 3.2 Correlation between clinical characteristics and CLNM

As shown in **Table 2**, sex, age, tumor size, enhancement intensity, and homogeneity of enhancement were significantly different between CLNM (+) and CLNM (−) subgroups of patients with PTC in both the training and validation cohorts (*p* < 0.05). The ROC curve showed that the best cutoff values of age and tumor size were 42 years with an AUC of 0.675 and 0.95 cm with an AUC of 0.709, respectively.

**Table 2:** Associations between the lymph node status and characteristics of patients in the training and validation cohorts.

Characteristics	Training cohort (*N* = 108)		*p*-value	Validation cohort (*N* = 108)		*p*-value
	CLNM (+)	CLNM (−)		CLNM (+)	CLNM (−)	
Sex			<0.001			0.038
Male	18 (37.5%)	4 (6.70%)		14 (26.4%)	6 (10.9%)	
Female	30 (62.5%)	56 (93.3%)		39 (73.6%)	49 (89.1%)	
Age (years)	39.63 ± 11.89	47.00 ± 11.99	0.002	39.85 ± 11.03	48.82 ± 10.18	<0.001
>42	13 (27.1%)	38 (63.3%)	<0.001	17 (32.1%)	38 (69.1%)	<0.001
≤42	35 (72.9%)	22 (36.7%)		36 (67.9%)	17 (30.9%)	
Size (cm)	1.33 ± 0.65	0.91 ± 0.37	<0.001	1.23 ± 0.59	0.86 ± 0.41	<0.001
≥0.95	32 (66.7%)	19 (31.7%)	<0.001	31 (58.5%)	17 (30.9%)	0.004
<0.95	16 (33.3%)	41 (68.3%)		22 (41.5%)	38 (69.1%)	
Location			0.948			0.679
Left lobe	21 (43.8%)	28 (46.7%)		22 (41.5%)	25 (45.5%)	
Right lobe	26 (54.2%)	31 (51.7%)		31 (58.5%)	30 (54.5%)	
Isthmus	1 (2.1%)	1 (1.7%)		0	0	
Margin			0.265			0.073
Regular	15 (31.3%)	25 (41.7%)		10 (18.9%)	4 (7.3%)	
Irregular	33 (68.8%)	35 (58.3%)		43 (81.1%)	51 (92.7%)	
Aspect ratio			0.116			0.823
>1	24 (50%)	39 (65.0%)		22 (41.5%)	24 (43.6%)	
≤1	24 (50%)	21 (35.0%)		31 (58.5%)	31 (56.4%)	
Calcification			0.174			0.172
Yes	43 (89.6%)	48 (80.0%)		41 (77.4%)	36 (65.5%)	
No	5 (10.4%)	12 (20.0%)		12 (22.6%)	19 (34.5%)	
Echogenicity			0.324			0.484
Hypoechoic	42 (87.5%)	57 (95.0%)		50 (94.3%)	54 (98.2%)	
Isoechoic	5 (10.4%)	2 (3.3%)		2 (3.8%)	1 (1.8%)	
Hyperechoic	1 (2.1%)	1 (1.7%)		1 (1.9%)	0	
Contact with the capsule			0.033			0.606
Yes	40 (83.3%)	39 (65.0%)		39 (73.6%)	38 (69.1%)	
No	8 (16.7%)	21 (35.0%)		14 (26.4%)	17 (30.9%)	
Enhancement intensity			0.002			0.004
Hypo-enhancement	19 (39.6%)	42 (70.0%)		20 (37.7%)	36 (65.5%)	
Iso-or hyperenhancement	29 (60.4%)	18 (30.0%)		33 (62.3%)	19 (34.5%)	
Enhancement patterns			0.455			0.956
Centripetal enhancement	27 (56.3%)	38 (63.3%)		34 (64.2%)	35 (63.6%)	
Hybrid enhancement	21 (43.8%)	22 (36.7%)		19 (35.85)	20 (36.4%)	
Homogeneity of enhancement			0.033			0.065
Homogeneous	10 (20.8%)	24 (40.0%)		15 (28.3%)	25 (45.5%)	
Heterogeneous	38 (79.2%)	36 (60.0%)		38 (71.7%)	30 (54.5%)	
Discontinuous capsular enhancement			0.161			0.250
Yes	18 (37.5%)	15 (25.0%)		29 (54.7%)	24 (43.6%)	
No	30 (62.5%)	45 (75.0%)		24 (45.2%)	31 (56.4%)	
Time of wash-in			0.547			0.032
Earlier	22 (45.8%)	31 (51.7%)		10 (18.9%)	3 (5.5%)	
Meantime and later	26 (54.2%)	29 (48.3%)		43 (81.1%)	52 (94.5%)	
Time of wash-out time			0.29			0.035
Earlier	13 (27.1%)	22 (36.7%)		28 (52.8%)	18 (21.8%)	
Meantime and later	35 (72.9%)	38 (63.3%)		25 (47.2%)	37 (67.3%)	
WIS	1.059 ± 0.976	1.037 ± 0.966	0.956	1.00 ± 0.50	0.74 ± 0.31	0.001
TP	1.072 ± 0.317	1.123 ± 0.509	0.466	1.29 ± 1.88	1.03 ± 0.42	0.799
PI	0.850 ± 0.325	0.802 ± 0.255	0.214	0.86 ± 0.37	0.69 ± 0.28	0.010
AUC	0.819 ± 0.329	0.825 ± 0.341	0.625	0.82 ± 0.40	0.68 ± 0.31	0.089

Contact with the capsule was associated with CLNM only in the training cohort (*p* = 0.033). Differences of WIS (*p* = 0.001) and PI (*p* = 0.010) between CLNM (+) and CLNM (−) groups were only observed in the validation cohort. There was no significant difference in other clinical characteristics. There were no differences of US and CEUS features between CLNM (+) and CLNM (−) patients with PTC (*p* > 0.05).

### 3.3 Feature selection and model construction

**Figure 2:**
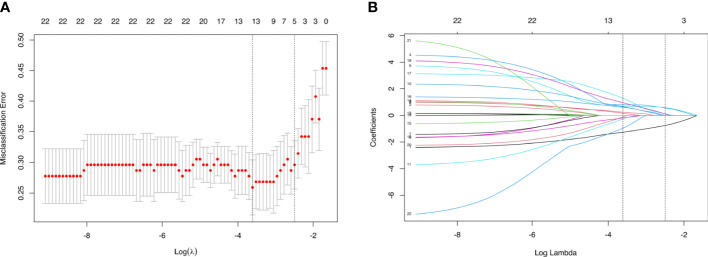
Parameters of prediction model selection using the LASSO logistic regression model in the training cohort. **(A)** The area under the receiver operating characteristic curve was plotted versus log (λ). **(B)** The features were profiled by the LASSO coefficient.

**Figure 3:**
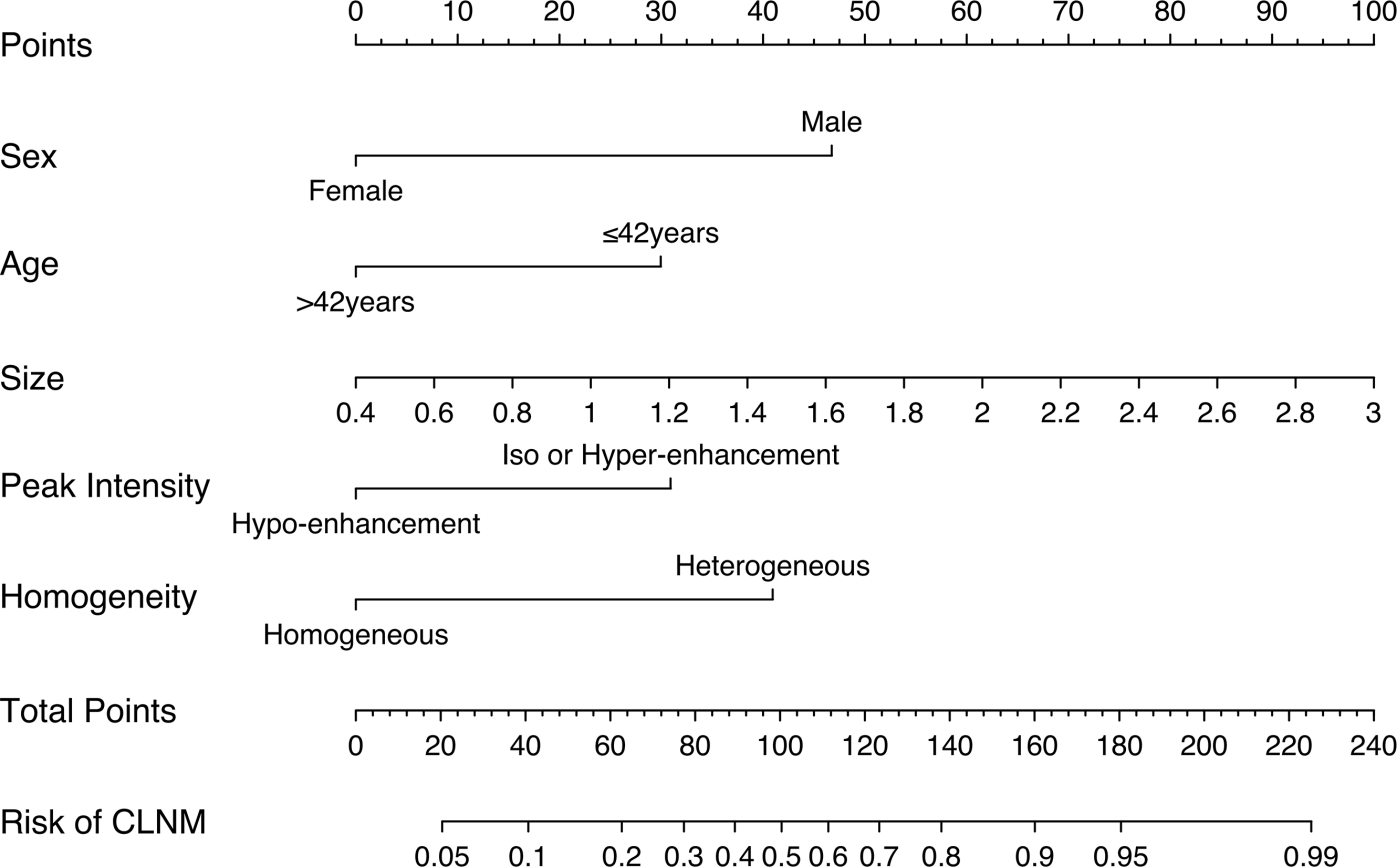
Nomogram of the prediction model to assess the risk of CLNM in patients with PTC.

In the training cohort, LASSO regression analysis was performed to select the useful predictive features for CLNM, including sex, age (≤42 years), size, and enhancement intensity (**Figure 2**). Multivariate logistic regression analysis revealed that sex, age (≤ 42 years), size, enhancement intensity, and homogeneity of enhancement were independent risk factors for CLNM (**Table 3**). All these risk factors were incorporated into the prediction model (**Figure 3**). This prediction model is displayed as a dynamic nomogram (https://clnmpredictionmodel.shinyapps.io/PTCCLNM/).

**Table 3:** Risk factors for cervical lymph node metastasis in the prediction model.

Intercept and variables	β	OR	95% CI	*p*-value
Sex	1.720	0.179	1.531–20.377	0.009
Age	1.102	3.010	1.097–8.263	0.032
Size	1.415	4.118	1.421–11.932	0.009
Peak Intensity	1.138	3.119	1.031–9.434	0.044
Degree of homogeneity	1.507	4.511	1.309–15.550	0.017
Intercept	−4.260	0.014	——	0.000

CI, confidence interval; β, regression coefficient; OR, odds ratio.

### 3.4 Evaluation and validation of the prediction models

**Figure 4:**
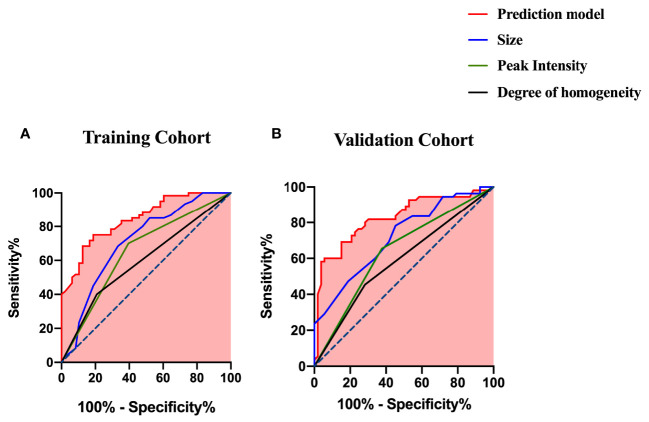
ROC curves of the prediction model and the single US and CEUS features for predicting CLNM **(A)** in the training cohort and **(B)** in the validation cohort.

The ROC curves of the prediction model and the single ultrasonic features in the training and validation cohorts are shown in **Figure 3**. There was good discrimination of the prediction model in the training (AUC: 0.844, 0.773–0.915) or validation (AUC: 0.827, 0.747–0.906) cohorts (**Table 4**). The AUC value of the prediction model was higher compared with any single US or CEUS feature (*p* < 0.05) (**Figure 4**).

**Table 4:** Performance of prediction model and the single US and CEUS features in the training and validation cohorts.

	Training cohort			Validation cohort		
	Sensitivity	Specificity	vAUC (95% CI)	Sensitivity	Specificity	AUC (95% CI)
Prediction model	81.3%	75.0%	0.844 (0.755–0.905)	77.4%	78.2%	0.827 (0.747–0.906)
Size	66.7%	68.3%	0.709 (0.609–0.810)	54.7%	78.2%	0.720 (0.625–0.814)
Peak INTENSITY	60.4%	70.0%	0.652 (0.547–0.757)	62.3%	65.5%	0.639 (0.533–0.744)
Degree of homogeneity	79.2%	40.0%	0.596 (0.489–0.703)	71.7%	45.5%	0.586 (0.478–0.693)

AUC, area under the curve; CI, confidence interval.

**Figure 5:**
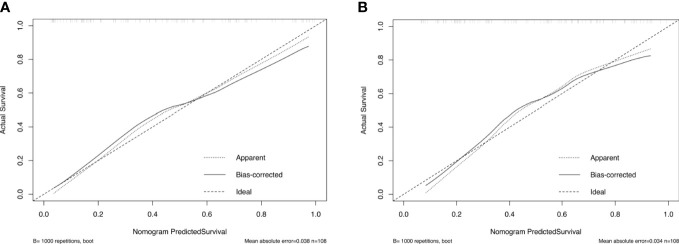
Calibration curves of the nomogram in the training **(A)** and validation **(B)** cohorts.

The calibration curve and Hosmer–Lemeshow test showed that the prediction model had good concordance in the training (*p* = 0.385) and validation (*p* = 0.285) cohorts (**Figure 5**).

### 3.5 Clinical use

**Figure 6:**
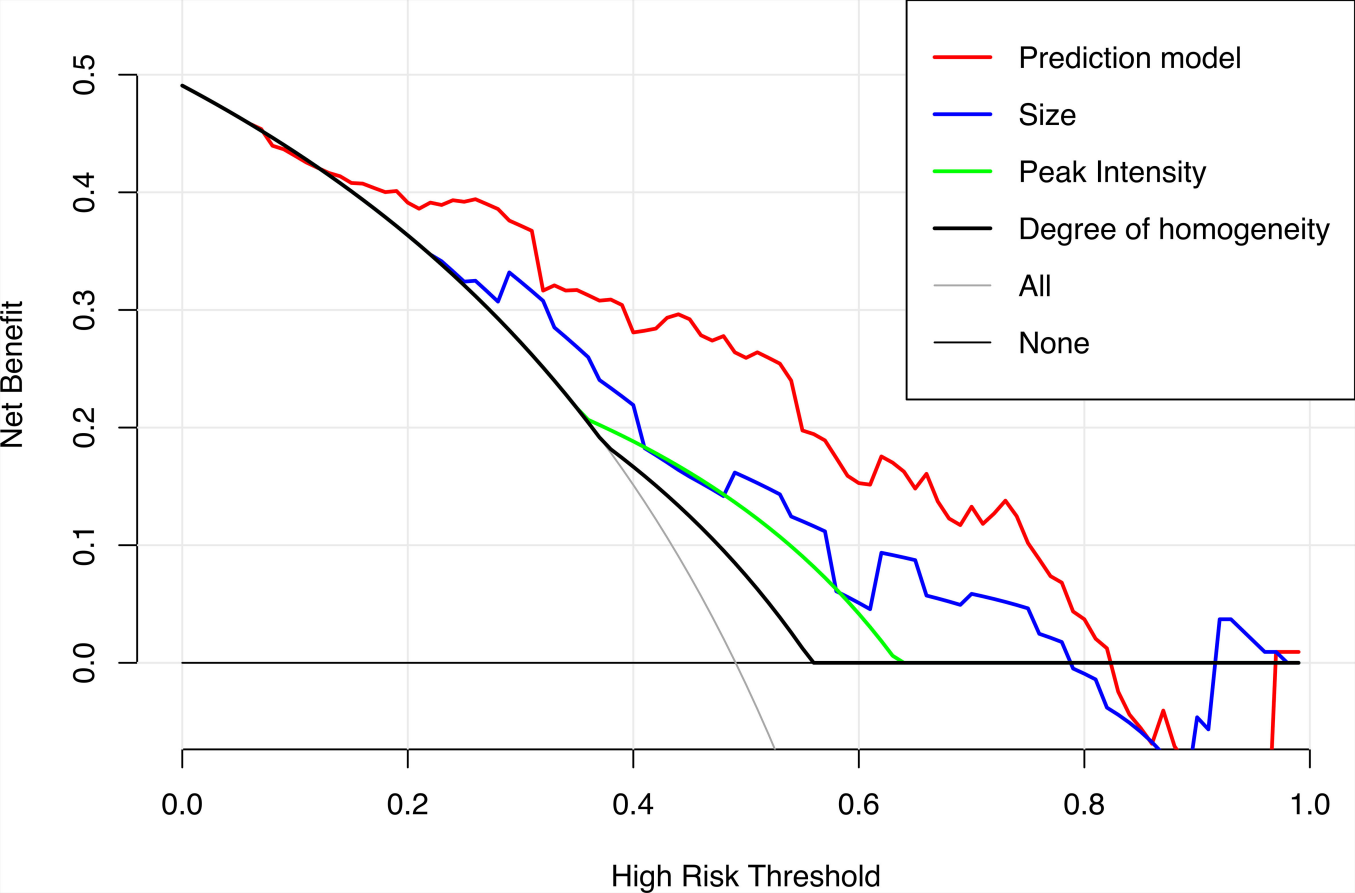
Decision curves of the prediction model and the single US and CEUS features in predicting CLNM for papillary thyroid carcinoma.

The DCA results of the prediction and clinical models are presented in **Figure 6**. Based on the DCA results, the prediction model has a higher clinical net benefit rate than the US or CEUS features alone in predicting CLNM when the threshold probability is between 7% and 82%.

**Figure 7:**
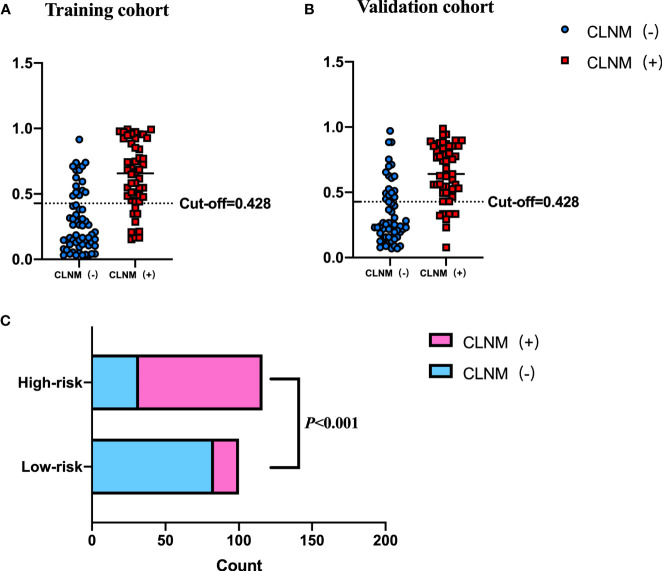
Performance of the cutoff value for predicting CLNM in patients with PTC. In **(A, B)**, the cutoff value of the Nomo-score performed well in the differential diagnosis of pN1 from pN0 in the training and validation cohorts, respectively. **(C)** The risk classification performance of the cutoff value is shown.

The cutoff value of the Nomo-score for the diagnosis of CLNM was ≥0.428. We divided patients into low-risk (100 patients) and high-risk groups (116 patients) using this cutoff value. Patients in the high-risk group were more likely to have CLNM (*p* < 0.001). CLNM (+) and CLNM (−) were discriminated well with a cutoff value of 0.428 in both the training and validation cohorts (**Figure 7**).

## 4 Discussion

In this study, we developed and validated a prediction model by combining US and CEUS features for evaluating the risk of CLNM in PTC patients. The prediction model presented as a dynamic nomogram was more convenient for clinical use, and it showed good diagnostic performance in both training and validation cohorts. A cutoff value derived from Nomo-score can be used for CLNM risk stratification in patients with PTC.

Only half of CLNM cases can be accurately detected by the conventional US (18–20). Some studies reported that the US features of PTC, such as calcification, taller than wide, and contact with the capsule, are related to CLNM (21, 22). However, these US features were not correlated with the CLNM in our study, likely owing to different definitions of US features among prior studies. For example, Tian et al. (23) reported that microcalcification within PTC was the strongest predictor for CLNM, and the type of microcalcification depended on its size. In contrast, we classified the microcalcification pattern as present or absent. Previous studies showed that larger tumor size was associated with an increased risk of CLNM (24). In our study, the binary variable of tumor size according to 0.95 cm was not related to CLNM in the LASSO logistic regression and multivariate logistic regression (*p* = 0.175). Considering that tumor size was associated with CLNM in both the training and validation cohorts, we incorporated tumor size into the prediction model as a continuous variable, and multivariate regression revealed a strong correlation with CLNM (OR: 4.118, *p* = 0.009).

Previous studies have shown that some US features were valuable for predicting CLNM in PTC patients, but the results were not consistent. In addition, it is difficult to predict CLNM with US alone. Therefore, we explored the value of multimodal US in the diagnosis of CLNM.

In this study, regarding the CEUS features of PTC, heterogeneous enhancement and iso- or hyperenhancement were associated with CLNM, which is consistent with some previous studies. PTC may destroy neovascular tissue when tumorous infiltration and metastasis occur, and it may cause perfusion defects as presented in heterogeneous enhancement (25). Of note, iso- or hyperenhancement is the strongest risk factor in the prediction model. Angiogenesis plays an important role in the process of tumor invasion and underlies the development, growth, and metastasis of tumor. Hyperenhancement indicates an abundant blood supply in the tumor microenvironment, which is associated with an increased risk for CLNM. Similar findings were found in high-grade breast tumors (26). As previously reported, some CEUS quantitative parameters can be applied to predict the risk of CLNM in PTC patients. We also analyzed quantitative parameters such as WIS, TP, PI, and AUC. In the training cohort, the PI and WIS were higher in patients with PTC who had CLNM than in patients without CLNM, but the difference was not statistically significant (*p* > 0.05). Tao et al. (14) reported that PI was an independent risk factor of CLNM. Considering PI value is likely related to tumor microvessel density, further showing angiogenesis’ important role in the development and metastases of tumors (27). However, limited sample in this study leads to the cautious interpretation of the results; thus, further study is required.

In the present study, younger age and male were related to a higher risk of CLNM in PTC patients, consistent with other studies (28, 29). Ning et al. (30) suggested that younger age may indicate a higher biological aggressiveness of tumor. Our study concluded that PTC patients who are less than 42 years old are prone to have CLNM, with an age cutoff value close to the value suggested by Tian et al. (≤40 years old). Accumulating evidence has shown an association between being male and a poor prognosis of PTC, but the results have been inconsistent (31–33).

We used LASSO and multivariate logistic regression in this study to select features. Our prediction model combining US, CEUS features, and clinical factors showed better diagnostic efficiency compared with the single ultrasonic imaging features in both training and validation cohorts. According to the DCA curve, the application of the prediction model could benefit patients more than a treat-none or treat-all strategy when the threshold probability was between 7% and 82%. We also established a risk stratification criterion based on the Nomo-score and showed that patients with a Nomo-score ≥ 0.428 were likely to have a higher incidence of CLNM. Therefore, this prediction model can be used to evaluate individuals preoperatively, and CLND was recommended for patients with a Nomo-score ≥ 0.428.

Our study has several limitations. First, the training cohort in this study was retrospective, and some bias inevitably exists. Second, the data might be affected by the different machines and probes used in the training and validation cohorts, but a ratio was used to reduce this effect. Third, this study needs to be further validated using a study with a larger sample size. Finally, all data were obtained from a single institution; thus, external validation in multicenter clinical trials is warranted.

In conclusion, a web-based dynamic nomogram based on US and CEUS features was constructed and showed a good performance in predicting the CLNM risk in PTC patients. This may be instrumental in refining surgery strategy in PTC patients.

## Data availability statement

The original contributions presented in the study are included in the article/supplementary material. Further inquiries can be directed to the corresponding author.

## Author contributions

QC performed the statistical work and wrote the first draft of the manuscript. YL, JL, and YS completed the data collection work. LQ and XH edited and revised the manuscript. All authors revised the manuscript critically and approved the final version of the manuscript.
